# Specific Biomarkers Involved in Sarcoidosis Diagnosis and Prognosis

**DOI:** 10.3390/ijms27146516

**Published:** 2026-07-22

**Authors:** Elena Matei, Viorica Pîslan (Zamfir), Victoria-Cristina Șuța, Georgeta Camelia Cozaru, Elena Danteș

**Affiliations:** 1 Center for Research and Development of the Morphological and Genetic Studies of Malignant Pathology, “Ovidius” University of Constanta, 145 Tomis Boulevard, 900591 Constanta, Romania; georgiana.cozaru@365.univ-ovidius.ro; 2 Clinical Department of Pneumology, The Pneumophthisiology Hospital of Constanta, 40 Santinelei Street, 900002 Constanta, Romania; viorica.zamfir@365.univ-ovidius.ro (V.P.); elena.dantes@365.univ-ovidius.ro (E.D.); 3 Medical Doctoral School, Medicine Faculty, “Ovidius” University of Constanta, 1 Universitatii Street, 900470 Constanta, Romania; 4 Medicine Faculty, “Ovidius” University of Constanta, 1 Universitatii Street, 900470 Constanta, Romania; cris_duminica@yahoo.com; 5 Internal Medicine and Rheumatology, Internal Medicine Section II, “Sf. Apostol Andrei” Emergency County Hospital, 145 Tomis Boulevard, 900591 Constanta, Romania; 6 Clinical Service of Pathology, “Sf. Apostol Andrei” Emergency County Hospital, 145 Tomis Boulevard, 900591 Constanta, Romania

**Keywords:** macrophages, T cells, natural killer cells, biomarkers, diagnosis, prognosis

## Abstract

Biomarker discovery in sarcoidosis mechanisms represents an innovative research tool. Inflammatory responses in sarcoidosis patients were analyzed by flow cytometry to highlight CD8a+CD45+, CD45+CD8a−, CD4+CD19+, CD4+CD19−, CD3+CD56+CD16+, CD56+CD16+, and CD16+CD56− as predictive biomarkers. In our study, monocyte-derived macrophage activation (M1 phenotype), represented by CD8a+CD45+ expression, is characteristic of granulomatous inflammation in active and progressive sarcoidosis and serves as a biomarker with very good accuracy and high specificity for establishing a diagnosis of sarcoidosis. As a prognostic biomarker, it is an independent predictive factor of disease progression, being negatively associated with applied treatments by sarcoidosis stage. The accumulation of activated CD4+ T-helper cells in sarcoid granulomas is responsible for the establishment of a pro-inflammatory phenotype and serves as a biomarker with good accuracy and high specificity for sarcoidosis diagnosis. As a prognostic biomarker, CD3+CD56+CD16+ (iNKT) is an independent predictor associated negatively with disease progression, being positively correlated with treatment applied by sarcoidosis stage. CD biomarker analysis, yielding predictive models with specificity for diagnosis and prognosis, is an important research tool for estimating disease progression in patients with different inflammatory response phenotypes across the stages of sarcoidosis.

## 1. Introduction

Sarcoidosis is a systemic granulomatous disease treated with immunomodulation to reduce inflammation and fibrosis. The mononuclear phagocyte system (MPS) recruits monocytes, which become macrophages in tissues, and these both, alongside tissue-resident macrophages, have important roles in development, tissue homeostasis, and inflammation [1]. Inflammatory monocyte-derived macrophages and tissue monocytes are phenotypically and functionally distinguished from resident macrophages in tissues. Recruited blood monocytes are a source of inflammatory macrophages, increasing tissue-resident macrophage proliferation by enhancing their self-renewal ability [2,3,4]. Macrophage differentiation from monocytes occurs in the tissue with the acquisition of a functional phenotype depending on microenvironmental signals [1].

Granuloma evolution involves pro-inflammatory cells, macrophages, CD4+ T-helper (Th) cells, B cells, dendritic cells (DCs), natural killer (NK) cells, and natural killer T (iNKT) cells [5,6,7]. Monocyte-derived macrophages are structurally involved in the cores of sarcoidosis granulomas [8]. In granuloma formation, macrophages acquire phenotypes as classically activated macrophages (M1) or activated macrophages (M2), with pro-inflammatory or pro-fibrotic capacities [1]. A transition from an M1 to M2 phenotype is characteristic of advanced stages of sarcoidosis [9,10,11].

Biomarkers play an important role in the diagnosis and prognosis of sarcoidosis, but their utility depends on the immune mechanisms underlying the various phenotypes. Understanding the phenotypes of sarcoidosis is necessary to develop promising tools to advance research on the disease [1].

In the early stages of sarcoidosis, CD4+ T lymphocytes accumulated in the alveolar cavities and interstitium. CD4, CD8, and CD19 lymphopenia in sarcoidosis patients suggest that lymphocytes are depleted in peripheral blood due to their increased infiltration in target organs [12]. Granuloma formation involves the accumulation of activated CD4+ T-helper lymphocytes [13]. Increased CD8+ T-cell counts are present in active and inactive chronic sarcoidosis [14]. Analyzing T cells from peripheral blood is a valuable approach to assessing sarcoidosis progression. In adaptive immunity, CD19 is a biomarker of lineage function used to identify activated B cells, which undergo rapid division and differentiation into antibody-producing plasma cells and memory B cells [15].

The balance of inflammatory cells from the innate and adaptive immune systems determines whether granulomatous inflammation remits, persists, or evolves into fibrosis [16,17]. Studies on natural killer T (iNKT) cells and natural killer (NK) cells involved in the immunopathogenesis of sarcoidosis are limited [18,19,20]. In sarcoidosis, iNKT cells play an immunoregulatory role, as decreased function contributes to enhanced T-cell activity involved in disease progression [21,22]. Natural killer T (iNKT, CD3+CD56+CD16+) cells represent a T-cell lineage that regulates cell-mediated immunity by expressing T-cell (CD3+) and NK (CD56+CD16+) receptors [19,23].

Biomarker discovery helps to understand the occurrence and progression of sarcoidosis, being useful for personalized treatment decisions [24,25]. Monocyte-derived macrophages are involved in granuloma formation in response to cytokines, chemokines, and other signaling proteins by activating their regulated pathways. Circulating biomarkers associated with sarcoidosis are involved in granuloma formation [16].

Because immune cell populations present considerable variety, our work highlights the discovery of new and alternative biomarkers that could be helpful in therapeutic strategies applied to patients with sarcoidosis. An appropriate choice of biomarkers in sarcoidosis leads to the optimal diagnosis of patients and appropriate decisions, being useful in therapeutic strategies and treatment. As biomarkers with optimal accuracy and specificity in sarcoidosis are limited, the possibility to study specific biomarkers involved in different stages of sarcoidosis, with different characteristics in terms of granuloma presence or not (active, progressive, or resolving sarcoidosis or non-active chronic sarcoidosis), offers the opportunity to improve diagnosis and prognosis for patients.

Inflammatory responses to different phenotypes across sarcoidosis stages were analyzed by flow cytometry methods to highlight CD45 monocyte-derived macrophages (CD45+NKR), CD8a cytotoxic T lymphocytes (CD8a+T), CD4 T-helper lymphocytes (CD4+ T-helper), CD19 monocyte-derived macrophages (CD19+; CD19M), natural killer T cells (CD3+CD56+CD16+; iNKT), natural killer cells (CD56+CD16+; NK), and intermediary monocytes (CD16+CD56−; CD16IM) as predictive biomarkers with specificity for diagnosis and prognosis, seeking to estimate disease progression and adverse outcomes, in combination with therapeutic strategies applied to patients with sarcoidosis.

## 2. Results

### 2.1. CD45 Monocyte-Derived Macrophages (CD45NKR) and CD8a Cytotoxic T (CD8aT) Lymphocytes Are Involved in Establishing Diagnosis and Prognosis Through Inflammatory Responses at Different Sarcoidosis Stages

**Figure 1:**
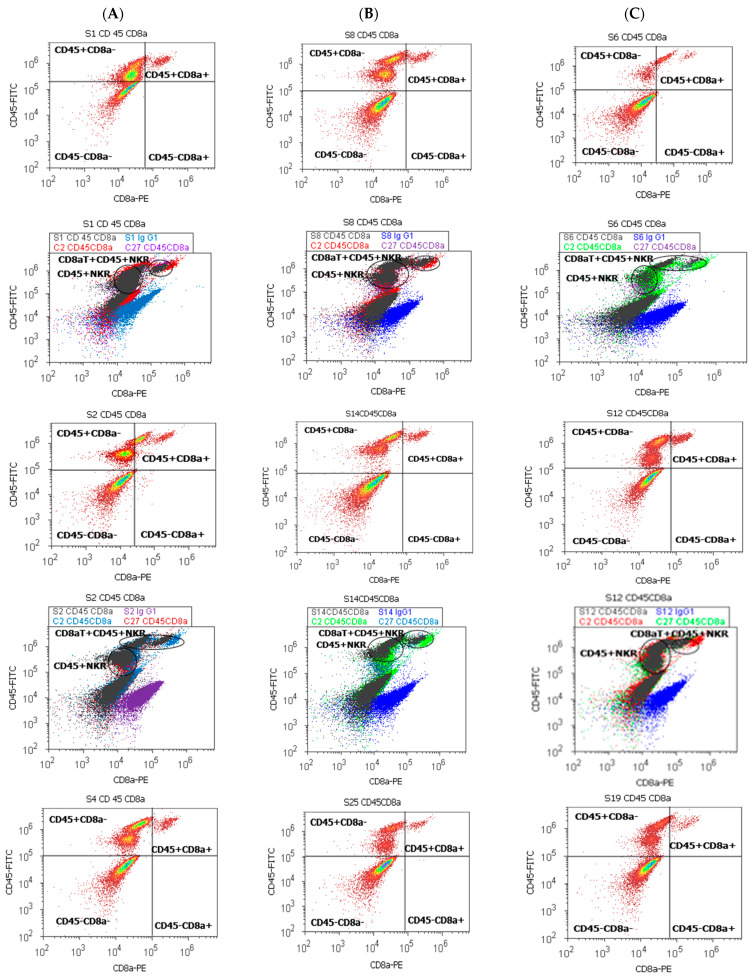

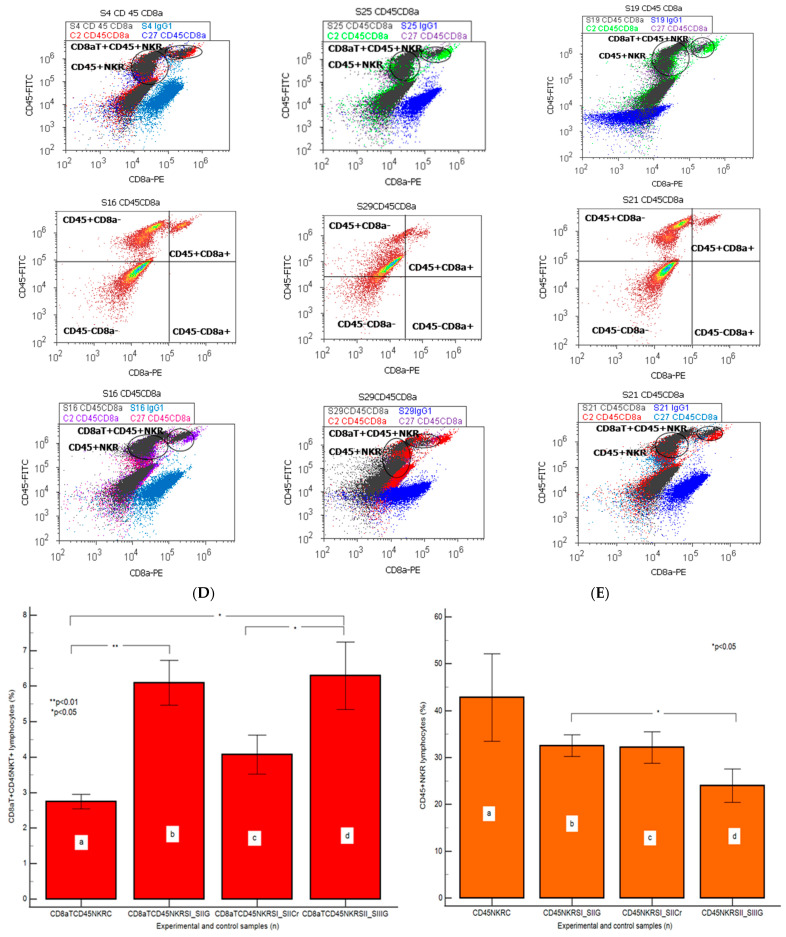
Inflammatory response in sarcoidosis highlighted by CD45 monocyte-derived macrophages (CD45NKR) and CD8a cytotoxic T (CD8aT) lymphocytes (**A**–**C**), obtained via the Attune Acoustic Focusing Cytometer, Life Technologies, Waltham, MA, USA, equipped with the Attune Cytometric Software v.1.2.5, 2010, Applied Biosystems, Waltham, MA, USA. ***CD45+CD8a+ (CD8aT+CD45+NKR):*** *(**A**) S1: 3.17%; S2: 11.81%; S4: 3.44%; S16: 5.55%; (**B**) S8: 3.91%; S14: 3.24%; S25: 1.37%; S29: 2.10%; (**C**) S6: 5.76%; S12: 3.78%; S19: 2.43%; S21: 2.67%. **CD45+CD8a− (CD45+NKR):** (**A**) S1: 48.23%; S2: 26.01%; S4: 39.26%; S16: 25.10%; (**B**) S8: 30.89%; S14: 19.58%; S25: 19.16%; S29: 25.99%; (**C**) S6: 4.77%; S12: 30.12%; S19: 60.63%; S21: 29.67%.* (**D**,**E**) Statistics of different types of inflammatory responses across sarcoidosis stages. ** p < 0.01 and * p < 0.05 indicate statistically significant differences between control and experimental samples via the Mann–Whitney test using the MedCalc Software, Ostend, Belgium. **Legend:** (**A**)—Active sarcoidosis, granulomatous inflammation, stage I–II (S1; S2; S4; S16); (**B**)—resolving sarcoidosis or non-active chronic sarcoidosis, without granulomatous inflammation, stage I–II (S8; S14; S25; S29); (**C**)—progressive sarcoidosis, active chronic sarcoidosis, granulomatous inflammation, stage II–III (S6; S12; S19; S21); (**a**) healthy patients (C; mean ± SD); (**b**) patients with active sarcoidosis, granulomatous inflammation, stage I–II (SI_IIG; mean ± SD); (**c**) patients with resolving sarcoidosis or non-active chronic sarcoidosis, without granulomatous inflammation, stage I–II (SI-II Cr; mean ± SD); (**d**) patients with progressive sarcoidosis, active chronic sarcoidosis, granulomatous inflammation, stage II–III (SII_SIIIG; mean ± SD); S—experimental sample; C-control sample; mouse IgG1 kappa isotype PE (IgG1)—negative control; C2; C27—control samples recovered from healthy patients.

In sarcoidosis, CD45+CD8a+ (CD8aT+CD45+NKR) and CD45+CD8a− (CD45+NKR) expression showed differences between experimental patient groups (A, B, C) in comparison to control samples recovered from healthy patients (C2, C27, Figure 1A–E, Table 1).

The pro-inflammatory cascade highlighted by CD8aT+CD45+NKR (CD45+CD8a+) expression presented statistically significant differences as a function of the sarcoidosis stage (SI_SIIG: 6.09 ± 3.21 (b); SI_SIICr: 4.08 ± 2.19 (c); SII_SIIIG:6.29 ± 3.00 (d) vs. C: 2.75 ± 0.58 (a), a, b *p* < 0.01; a, d, *p* < 0.05; c, d, *p* < 0.05, Figure 1D, Table 1).

CD45 natural killer receptors (CD45+NKR; CD45+CD8a−) had variable patterns, being involved in different patient groups across sarcoidosis stages (SI_SIIG: 32.54 ± 11.71 (b), SI_SIICr: 32.17 ± 13.42 (c); SII_SIIIG: 24.00 ± 11.37 (d); vs. C: 42.79 ± 26.39 (a), a, b, c, d, *p* ≥ 0.05; b, d, *p* < 0.05, Figure 1E, Table 1).

**Figure 2:**
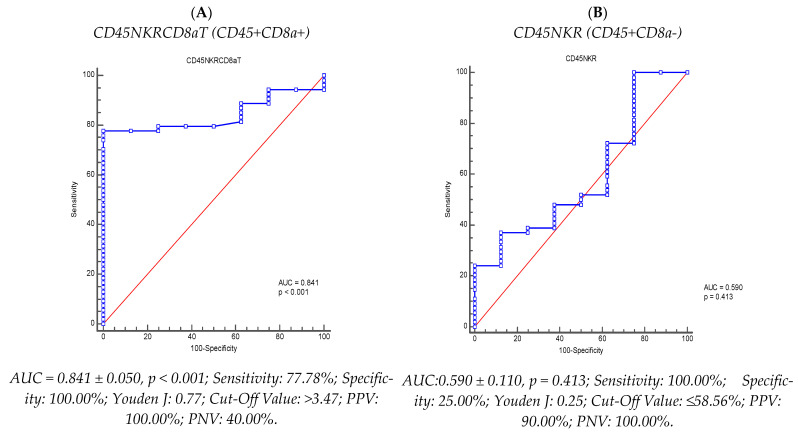

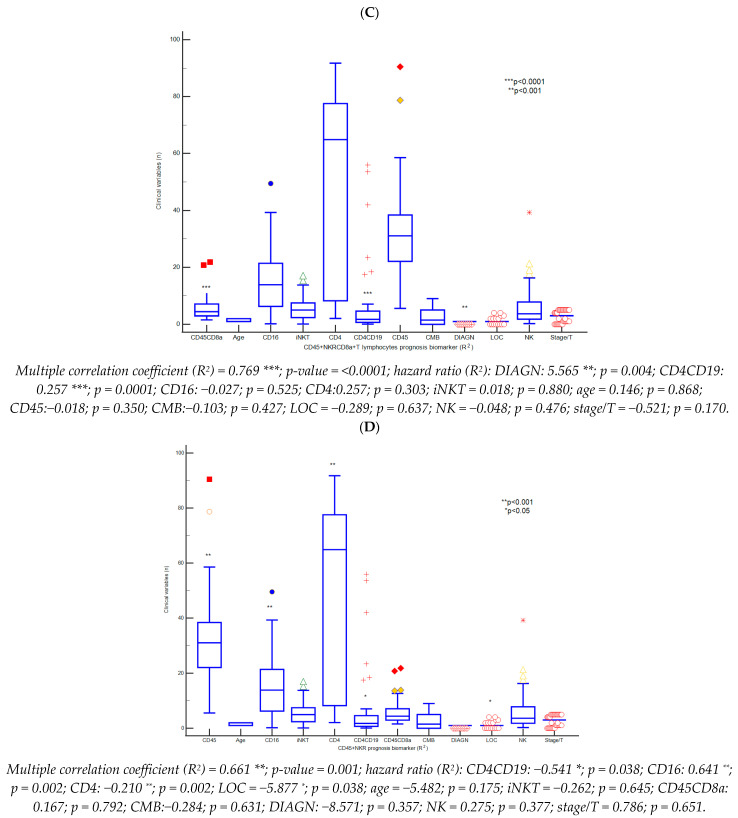
Receiver operating characteristic (ROC) analysis of CD biomarkers (**A**,**B**) in sarcoidosis. (**C**,**D**) Prognostic biomarkers (CD45+NKRCD8a+T; CD45+NKR) in sarcoidosis. *** p < 0.0001, ** p < 0.001, and * p < 0.05 indicate statistically significant differences between control and experimental samples as represented by least-squares multiple regression (R^2^) using the MedCalc v20.111 Software Ltd., Ostend, Belgium. **Legend:** NKR—natural killer receptor; T— T lymphocyte; AUC—area under the curve; PPV—positive value prediction; PNV—negative value prediction; CMB—comorbidities; DIAGN—diagnosis; LOC—localization; stage/T—associated treatment by sarcoidosis stage.

In establishing a diagnosis of sarcoidosis, predictive models, such as receiver operating characteristic (ROC) analysis of CD biomarkers, are important in estimating the patient’s risk of unfavorable outcomes. A single ROC curve was generated for the CD45NKRCD8aT (CD45+CD8a+) and CD45NKR (CD45+CD8a−) biomarkers with two overlapping distributions (control and experimental, Figure 2A,B).

The discrimination threshold (AUC = 0.841 ± 0.050, *p* < 0.001) for CD45NKRCD8aT expression, with sensitivity of 77.78% and specificity of 100.00%, indicated a biomarker with very good accuracy and high specificity in establishing a sarcoidosis diagnosis (Figure 2A).

For the CD45NKR biomarker, the discrimination threshold (AUC = 0.590 ± 0.110, *p* = 0.413), with sensitivity of 100.00% and specificity of 25.00%, indicated modest accuracy and a non-specific biomarker in sarcoidosis diagnosis (Figure 2B).

In sarcoidosis patients, specific biomarkers were associated with clinical variables using least-squares multiple regression (R^2^), highlighting predictive factors involved in sarcoidosis prognosis (Figure 2C,D).

The CD45+NKRCD8a+T (CD45+CD8a+) biomarker is an independent predictor (R^2^ = 0.769; *p* < 0.0001) of disease progression and adverse outcomes (diagnosis: R^2^ = 5.565, *p* = 0.004) and positively correlated with pro-inflammatory responses regarding CD4+CD19+ expression (R^2^ = 0.257, *p* = 0.0001, Figure 2C). CD45+NKRCD8a+T is a prognostic biomarker for disease progression, being negatively associated with the treatment response by sarcoidosis stage (R^2^ = −0.521, *p* = 0.170, Figure 2C).

The CD45+NKR (CD45+CD8a−) biomarker represents an independent predictive factor (R^2^ = 0.661; *p* = 0.001) in sarcoidosis patients, positively correlated with pro-inflammatory responses regarding CD16+CD56− monocytes (R^2^ = 0.641, *p* = 0.002) and negatively correlated with CD4+T-helper lymphocytes (R^2^ = −0.210, *p* = 0.002), CD4+CD19+ expression (R^2^ = −0.541, *p* = 0.038), and localization (R^2^ = −5.877, *p* = 0.038). CD45+NKR represents a prognostic biomarker, associated negatively with diagnosis (R^2^ = −8.571, *p* = 0.357) and positively with the applied treatment by sarcoidosis stage (R^2^ = 0.786, *p* = 0.651, Figure 2D).

### 2.2. CD4 T-Helper (CD4Th) Lymphocytes and CD19 Monocyte-Derived Macrophages (CD19M) Are Involved in Establishing Diagnosis and Prognosis Through Inflammatory Responses at Different Sarcoidosis Stages

**Figure 3:**
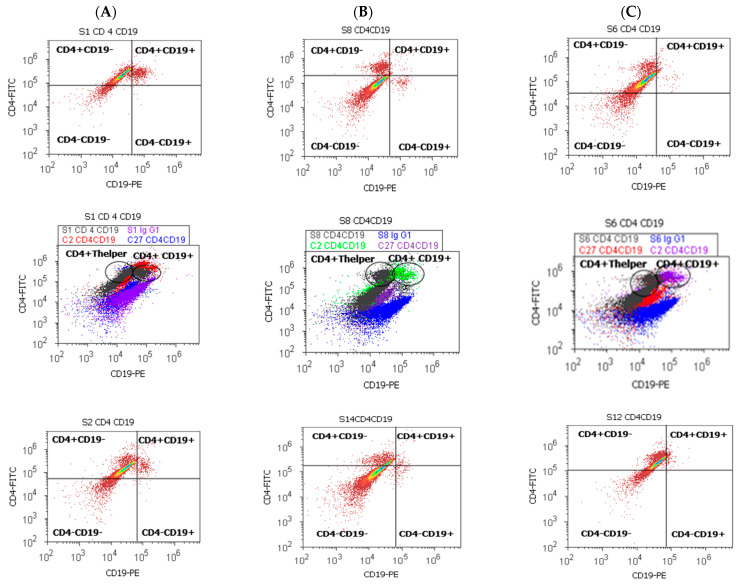

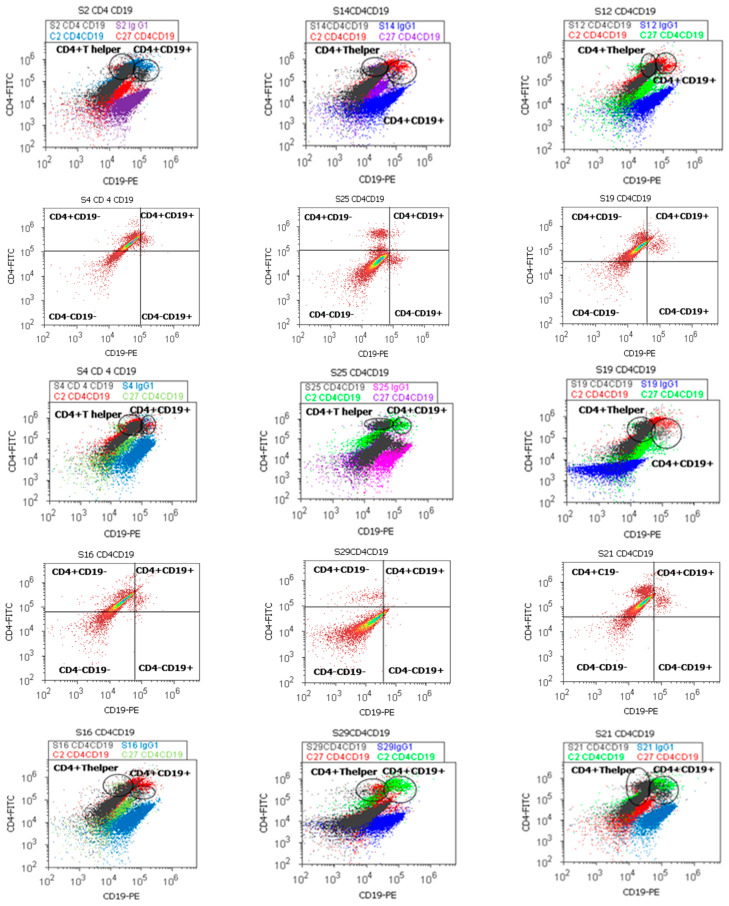

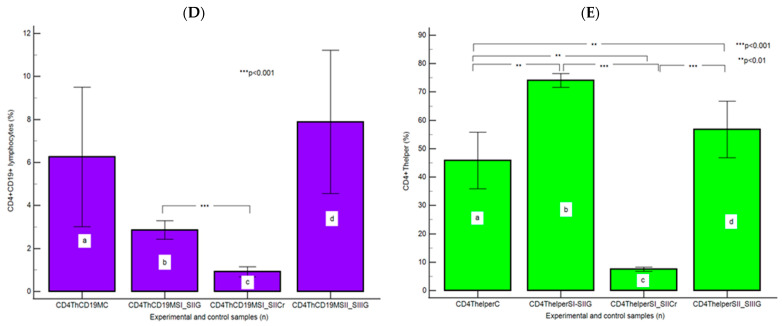
Pro-inflammatory reactions in sarcoidosis, highlighted by CD4+ T-helper (CD4Th) lymphocytes and CD19 monocyte-derived macrophages (CD19M, **A**–**C**), obtained via the Attune Acoustic Focusing Cytometer, Life Technologies, Waltham, MA, USA, equipped with the Attune Cytometric Software v.1.2.5, 2010, Applied Biosystems, Waltham, MA, USA. ***CD4+CD19+:*** *(***A***) S1: 7.87%; S2: 3.00%; S4: 1.50%; S16: 1.24%; (***B***) S8: 0.96%; S14: 1.89%; S25: 0.29%; S29: 0.15%; (***C***) S6: 1.47%; S12: 5.40%; S19: 2.52%; S21: 12.30%; **CD4+CD19− (CD4+ T helper):** (***A***) S1: 75.85%; S2: 52.33%; S4: 68.26%; S16: 41.30%; (***B***) S8: 9.41%; S14: 17.86%; S25: 4.59%; S29: 1.32%; (***C***) S6: 67.49%; S12: 62.17%; S19: 76.19%; S21: 73.72%.* (**D**,**E**) Statistics of different types of inflammatory responses across sarcoidosis stages. *** p < 0.001 and ** p < 0.01 indicate statistically significant differences between control and experimental samples via Mann–Whitney test using the MedCalc Software, Ostend, Belgium. **Legend:** (**A**)—Active sarcoidosis, granulomatous inflammation, stage I–II (S1; S2; S4; S16); (**B**)—resolving sarcoidosis or non-active chronic sarcoidosis, without granulomatous inflammation, stage I–II (S8; S14; S25; S29); (**C**)—progressive sarcoidosis, active chronic sarcoidosis, granulomatous inflammation, stage II–III (S6; S12; S19; S21); (**a**) healthy patients (C; mean ± SD); (**b**) patients with active sarcoidosis, granulomatous inflammation, stage I–II (SI_IIG; mean ± SD); (**c**) patients with resolving sarcoidosis or non-active chronic sarcoidosis, without granulomatous inflammation, stage I–II (SI-II Cr; mean ± SD); (**d**) patients with progressive sarcoidosis, active chronic sarcoidosis, granulomatous inflammation, stage II–III (SII_SIIIG; mean ± SD); S—experimental sample; C-control sample; mouse IgG1 kappa isotype PE (IgG1)—negative control; C2; C27—control samples recovered from healthy patients.

A pro-inflammatory phenotype, highlighted by CD4+ T-helper lymphocyte and CD19 monocyte-derived macrophage (CD19+; CD19M) immune reactions involved in permanent granulomatous formation, was observed in active sarcoidosis in stage I–II (A) or active chronic sarcoidosis, recognized as progressive sarcoidosis, in stage II–III (C), and without granulomas in resolving sarcoidosis or non-active chronic sarcoidosis, stage I–II (B, Figure 3A–C).

The development and evolution of granulomas involve CD4+ T-helper lymphocytes and CD19 monocyte-derived macrophages (CD4+CD19+; CD4ThCD19M), which showed significant differences between experimental groups (SI_SIIG: 2.86 ± 2.19 (b); SI_SIICr: 0.91 ± 1.00 (c); SII_SIIIG: 7.88 ± 11.55 (d) vs. C: 6.26 ± 9.18 (a), b, c, *p* < 0.001; a, b, c, d, *p* ≥ 0.05, Figure 3D, Table 1).

Systemic granulomatous responses, represented predominantly by CD4+ T-helper lymphocytes (CD4+CD19−; CD4+ T helper), presented significant differences between the experimental and control groups (SI_SIIG: 74.05 ± 12.61 (b); SI_SIICr: 7.54 ± 3.30 (c); SII_SIIIG: 56.83 ± 34.60 (d) vs. C: 45.86 ± 28.24 (a), a, b, c, d, *p* < 0.01; b, c, *p* < 0.001; c, d, *p* < 0.001; Figure 3E, Table 1).

**Figure 4:**
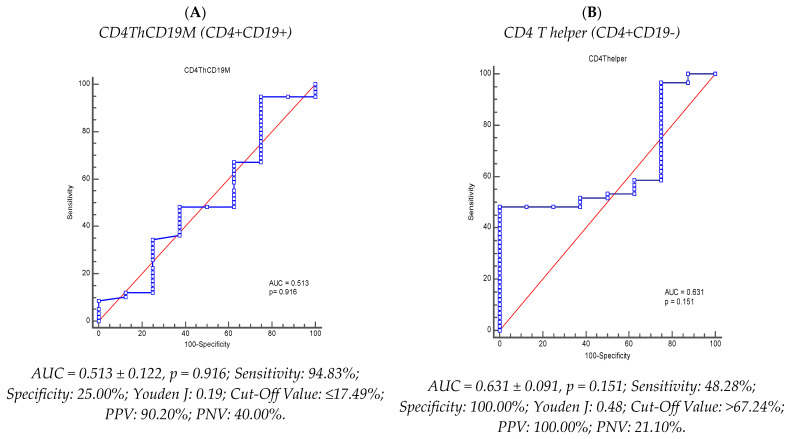

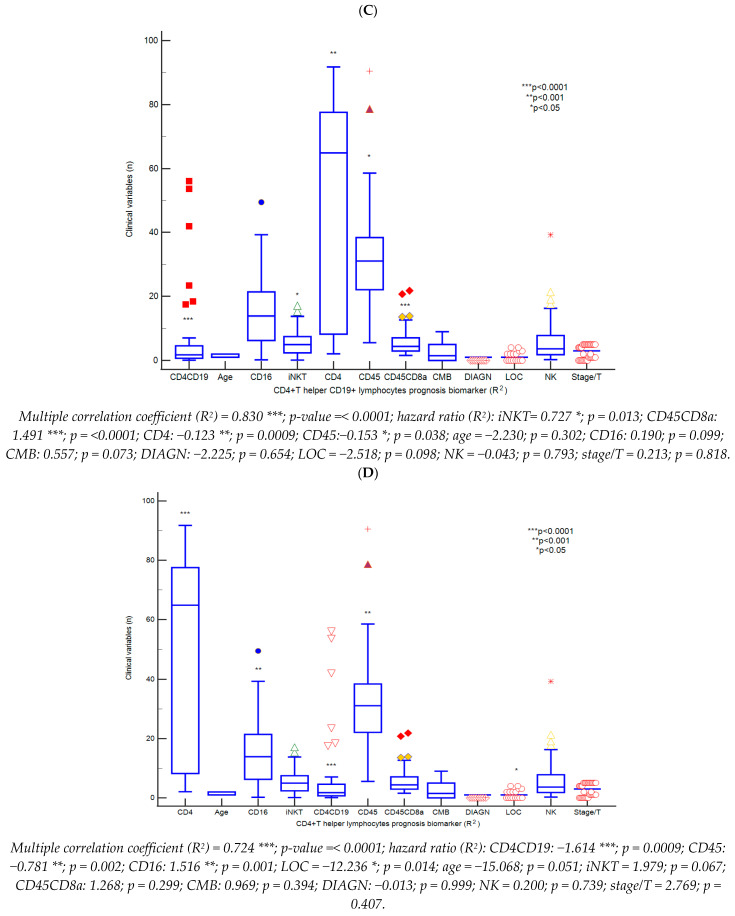
Receiver operating characteristic (ROC) analysis of CD biomarkers (**A**,**B**) in sarcoidosis. (**C**,**D**) Prognostic biomarkers (CD4+ T helper CD19+; CD4+ T helper) in sarcoidosis. *** p < 0.0001, ** p < 0.001, and * p < 0.05 indicate statistically significant differences between control and experimental samples as represented by least-squares multiple regression (R^2^) using the MedCalc v20.111 Software Ltd., Ostend, Belgium. **Legend:** NKR—natural killer receptor; T—lymphocyte; AUC—area under the curve; PPV—positive value prediction; PNV—negative value prediction; CMB—comorbidities; DIAGN—diagnosis; LOC—localization; stage/T—associated treatment by sarcoidosis stage.

The predictive model consisting of the ROC curve for the CD4+CD19+ (CD4ThCD19M) biomarker presents a discrimination threshold (AUC = 0.513 ± 0.122, *p* = 0.916) with modest accuracy, without specificity for sarcoidosis diagnosis, represented by sensitivity of 94.83% and specificity of 25.00% (Figure 4A).

The discrimination threshold (AUC = 0.631 ± 0.091, *p* = 0.151) with sensitivity of 48.28% and specificity of 100.00% for CD4+ T-helper (CD4+CD19−) expression indicated a biomarker with good accuracy, with high specificity for sarcoidosis diagnosis (Figure 4B).

As predictive factors involved in sarcoidosis prognosis, we analyzed the CD4+ T-helper CD19+ and CD4+ T-helper biomarkers via least-squares multiple regression (R^2^, Figure 4C,D).

CD4+CD19+ lymphocytes represent an independent predictive factor (R^2^ = 0.830; *p* < 0.0001) in sarcoidosis patients, being positively correlated with natural killer T-cell (iNKT, R^2^ = 0.727, *p* = 0.013) and CD45+NKRCD8a+T expression (R^2^ = 1.491, *p* < 0.0001) and negatively correlated with the pro-inflammatory response regarding CD4+ T-helper lymphocytes (R^2^ = −0.123, *p* = 0.0009) and CD45+NKR (R^2^ = −0.153, *p* = 0.038, Figure 4C). CD4+CD19+ expression represents a prognostic biomarker, being associated negatively with diagnosis (R^2^ = −2.225, *p* = 0.654) and with treatment applied by sarcoidosis stage (R^2^ = −0.213, *p* = 0.818, Figure 4C).

In sarcoidosis, granuloma formation is accompanied by the accumulation of activated CD4+ T-helper cells (CD4+CD19−), which are responsible for the induction of a pro-inflammatory phenotype. In sarcoidosis patients, CD4+ T-helper expression represents an independent predictive factor (R^2^ = 0.724; *p* < 0.0001), being positively correlated with CD16+CD56− intermediary monocytes (R^2^ = 1.516, *p* = 0.001) and negatively correlated with CD4+CD19+ lymphocytes (R^2^ = −1.614, *p* = 0.0009), CD45+NKR (R^2^ = −0.781, *p* = 0.020), and localization (R^2^ = −12.236, *p* = 0.014, Figure 4D). CD4+ T-helper expression represents a prognostic biomarker, being associated negatively with diagnosis (R^2^ = −0.013, *p* = 0.999) and positively with the applied treatment by sarcoidosis stage (R^2^ = 2.769, *p* = 0.407, Figure 4D).

### 2.3. Natural Killer T Cell (CD3+CD56+CD16+), Natural Killer Cell (CD56+CD16+), and Intermediary Monocyte (CD16+CD56−) Expressions Is Involved in Establishing Diagnosis and Prognosis via Inflammatory Responses at Different Sarcoidosis Stages

**Figure 5:**
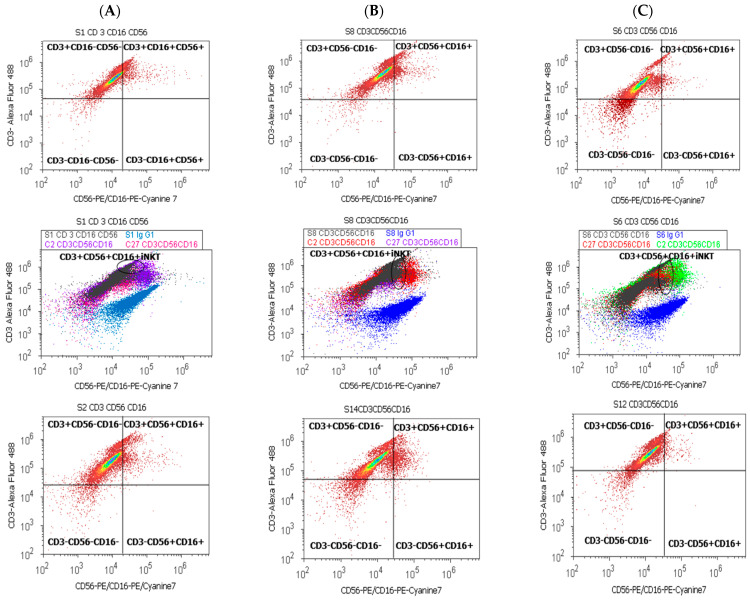

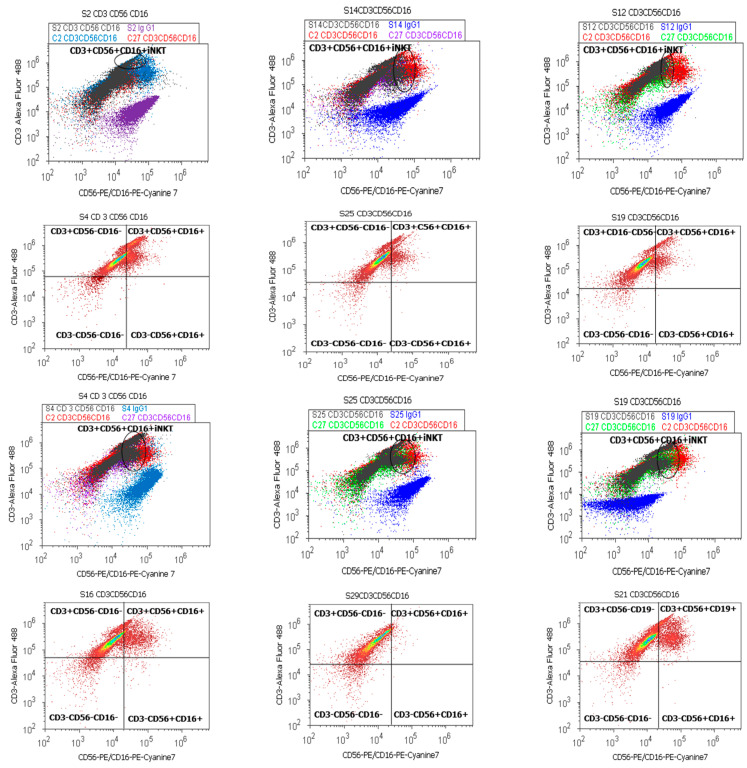

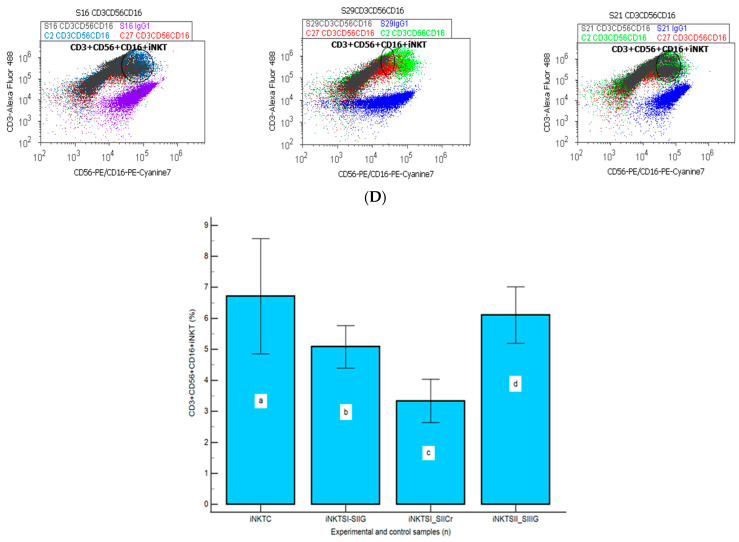
Natural killer T cell (CD3+CD56+CD16+; iNKT) expression in sarcoidosis (**A**–**C**), assessed using the Attune Acoustic Focusing Cytometer, Life Technologies, Waltham, MA, USA, equipped with the Attune Cytometric Software v.1.2.5, 2010, Applied Biosystems, Waltham, MA, USA. ***CD3+CD56+CD16+ (iNKT):*** *(***A***) S1: 9.24%; S2: 4.23%; S4: 15.64%; S16: 7.99%;* (**B**) *S8: 12.07%; S14: 8.64%; S25: 10.78%; S29: 2.36%;* (**C**) *S6: 3.06%; S12: 4.38%; S19: 6.38%; S21: 21.89%.* (**D**) Statistics of different types of inflammatory responses across sarcoidosis stages. **Legend:** (**A**)—Active sarcoidosis, granulomatous inflammation, stage I–II (S1; S2; S4; S16); (**B**)—resolving sarcoidosis or non-active chronic sarcoidosis, without granulomatous inflammation, stage I–II (S8; S14; S25; S29); (**C**)—progressive sarcoidosis, active chronic sarcoidosis, granulomatous inflammation, stage II–III (S6; S12; S19; S21); (**a**) healthy patients (C; mean ± SD); (**b**) patients with active sarcoidosis, granulomatous inflammation, stage I–II (SI_IIG; mean ± SD); (**c**) patients with resolving sarcoidosis or non-active chronic sarcoidosis, without granulomatous inflammation, stage I–II (SI-II Cr; mean ± SD); (**d**) patients with progressive sarcoidosis, active chronic sarcoidosis, granulomatous inflammation, stage II–III (SII_SIIIG; mean ± SD); S—experimental sample; C-control sample; mouse IgG1 kappa isotype PE (IgG1)—negative control; C2; C27—control samples recovered from healthy patients.

The inflammatory response leading to granuloma formation involves both the innate and adaptive immune systems. Understanding the role of T cells in sarcoidosis has led to the development of new therapies targeting the pathogenic T-lymphocyte phenotype (CD3+CD56+CD16+; iNKT in Figure 5A–C).

NKT expression (CD3+CD56+CD16+; iNKT) in disease pathogenesis showed differences between the experimental and control groups (iNKT: SI_SIIG: 5.07 ± 3.47 (b); SI_SIICr: 3.33 ± 2.80 (c); SII_SIIIG: 6.10 ± 3.16 (d) vs. C: 6.71 ± 5.26 (a), a, b, c, d, *p* ≥ 0.05, Figure 5D, Table 1).

**Figure 6:**
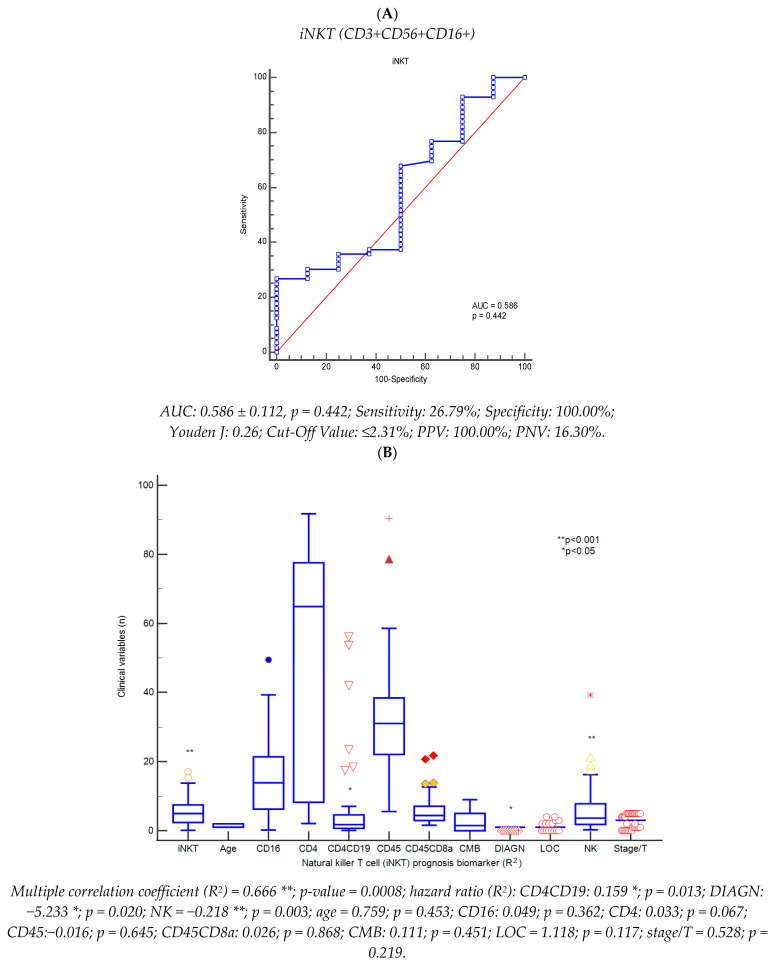
Receiver operating characteristic (ROC) analysis of CD biomarkers (**A**) in sarcoidosis. (**B**) Prognostic biomarker (iNKT; CD3+CD56+CD16+) in sarcoidosis. ** p < 0.001 and * p < 0.05 indicate statistically significant differences between control and experimental samples as represented by least-squares multiple regression (R^2^) using the MedCalc v20.111 Software Ltd., Ostend, Belgium. **Legend:** NKR—natural killer receptor; T—T lymphocyte; AUC—area under the curve; PPV—positive value prediction; PNV—negative value prediction; CMB—comorbidities; DIAGN—diagnosis; LOC—localization; stage/T—associated treatment by sarcoidosis stage.

The discrimination threshold (AUC = 0.586 ± 0.112, *p* = 0.442) with sensitivity of 26.79% and specificity of 100.00% for CD3+CD56+CD16+ (iNKT) expression indicated a biomarker with modest accuracy, with high specificity for sarcoidosis diagnosis (Figure 6A). In sarcoidosis, the expression of iNKT (CD3+CD56+CD16+) was associated with clinical variables, as assessed by least-squares multiple regression (R^2^), aiming to identify prognostic factors (Figure 6B).

The CD3+CD56+CD16+ (iNKT) biomarker is an independent predictive factor (R^2^ = 0.666; *p* = 0.0008) involved in disease progression and adverse outcomes (diagnosis: R^2^ = −5.233, *p* = 0.020), being positively correlated with pro-inflammatory responses regarding CD4+CD19+ lymphocytes (R^2^ = 0.159, *p* = 0.013) and NK cells (R^2^ = 0.218, *p* = 0.003, Figure 6B).

CD3+CD56+CD16+ expression serves as a prognostic biomarker, being negatively associated with disease progression (R^2^ = −5.233, *p* = 0.020) and positively correlated with the treatment response across sarcoidosis stages (R^2^ = 0.528, *p* = 0.219, Figure 6B).

**Figure 7:**
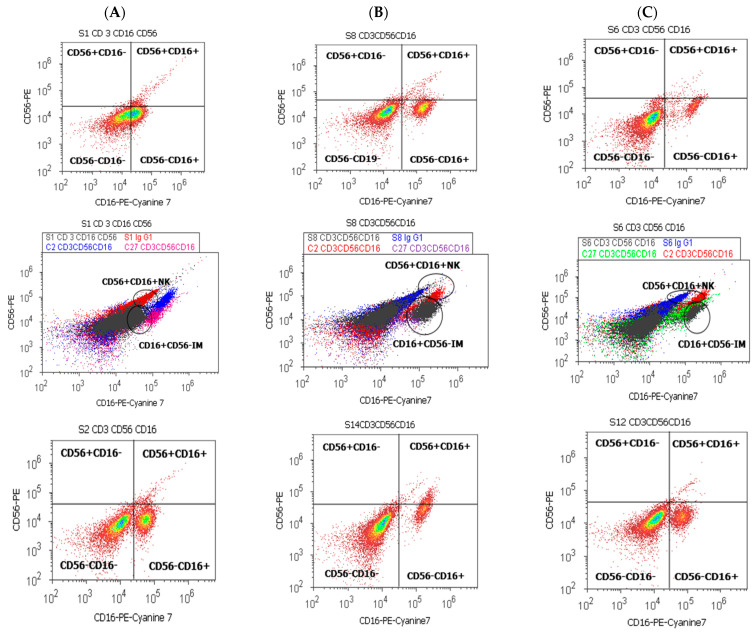

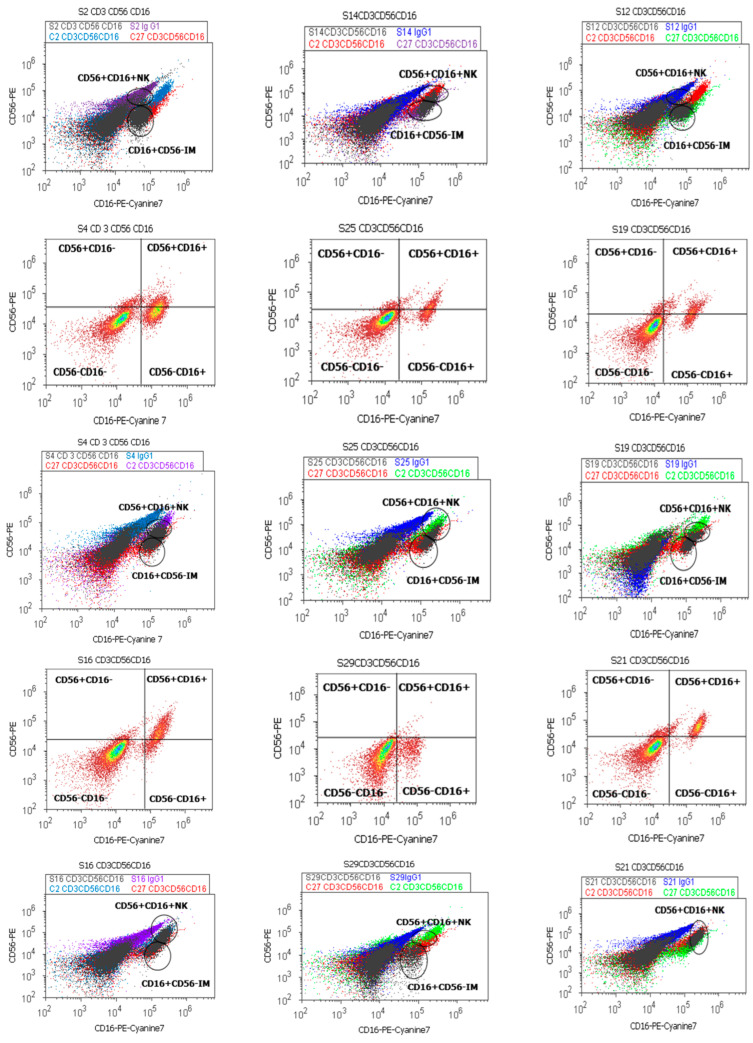

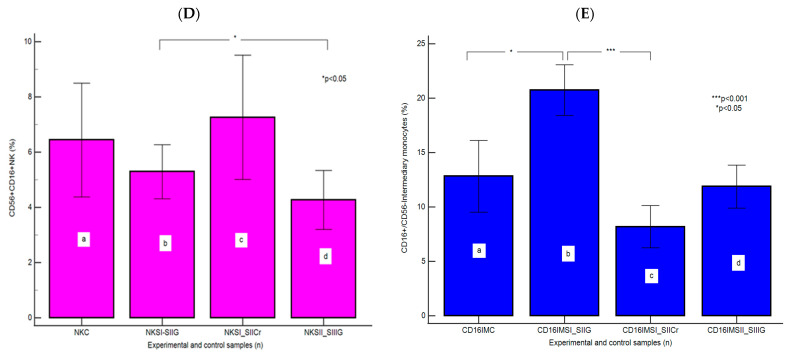
Natural killer cell (CD56+CD16+; NK) and intermediary monocyte (CD16+CD56−; CD16-IM) expression in sarcoidosis (**A**–**C**) was assessed via the Attune Acoustic Focusing Cytometer, Life Technologies, Waltham, MA, USA, equipped with the Attune Cytometric Software v.1.2.5, 2010, Applied Biosystems, Waltham, MA, USA. ***CD56+CD16+ (NK):*** *(***A***) S1: 3.19%; S2: 1.37%; S4: 7.18%; S16: 10.62%; (***B***) S8: 0.73%; S14: 3.46%; S25: 3.81%; S29: 0.12%; (***C***) S6: 1.90%; S12: 0.49%; S19: 3.48%; S21: 15.24%; **CD16+CD56− (CD16+CD56−IM):** (***A***) S1: 59.50%; S2: 26.48%; S4: 25.16%; S16: 7.03%; (***B**) *S8: 23.22%; S14: 9.45%; S25: 8.29%; S29: 7.28%; (***C***) S6: 8.37%; S12: 16.57%; S19: 7.46%. (**D**,**E**)* Statistics of different types of inflammatory responses across sarcoidosis stages. *** p < 0.001 and * p < 0.05 indicate statistically significant differences between control and experimental samples via the Mann–Whitney test using the MedCalc Software, Ostend, Belgium. **Legend:** (**A**)—Active sarcoidosis, granulomatous inflammation, stage I–II (S1; S2; S4; S16); (**B**)—resolving sarcoidosis or non-active chronic sarcoidosis, without granulomatous inflammation, stage I–II (S8; S14; S25; S29); (**C**)—progressive sarcoidosis, active chronic sarcoidosis, granulomatous inflammation, stage II–III (S6; S12; S19; S21); (**a**) healthy patients (C; mean ± SD); (**b**) patients with active sarcoidosis, granulomatous inflammation, stage I–II (SI_IIG; mean ± SD); (**c**) patients with resolving sarcoidosis or non-active chronic sarcoidosis, without granulomatous inflammation, stage I–II (SI-II Cr; mean ± SD); (**d**) patients with progressive sarcoidosis, active chronic sarcoidosis, granulomatous inflammation, stage II–III (SII_SIIIG; mean ± SD); S—experimental sample; C-control sample; mouse IgG1 kappa isotype PE (IgG1)—negative control; C2; C27—control samples recovered from healthy patients.

NK cells (CD56+CD16+; NK) and intermediary monocytes (CD16+CD56−; CD16IM) were presented in Figure 7A–C. Natural killer cell (CD56+CD16+; NK) and intermediary monocyte (CD16+CD56−; CD16IM) expression in pathogenesis presented differences between the experimental and control groups (NK: SI_SIIG: 5.29 ± 4.97 (b); SI_SIICr: 7.26 ± 9.55 (c); SII_SIIIG: 4.26 ± 3.71 (d) vs. C: 6.44 ±5.81 (a), a, c, *p* ≥ 0.05; b, d, *p* < 0.05, Figure 7D; CD16IM: SI_SIIG: 20.74 ± 11.84 (b); SI_SIICr: 8.19 ± 8.28 (c); SII_SIIIG: 11.88 ± 6.84 (d) vs. C: 12.82 ± 9.32 (a), a, b, *p* < 0.05, b, c, *p* < 0.001, Figure 7E, Table 1).

**Figure 8:**
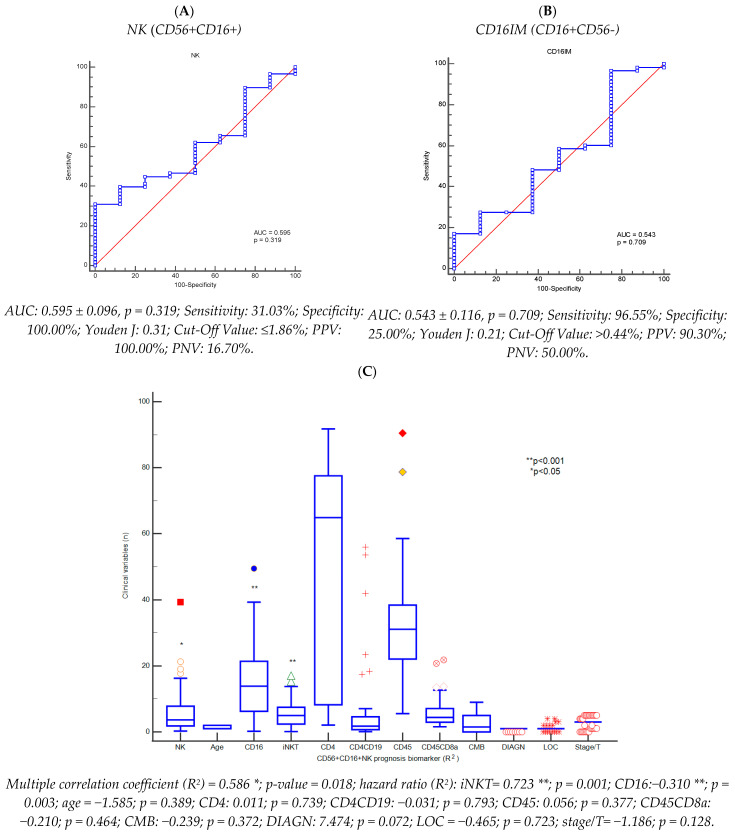

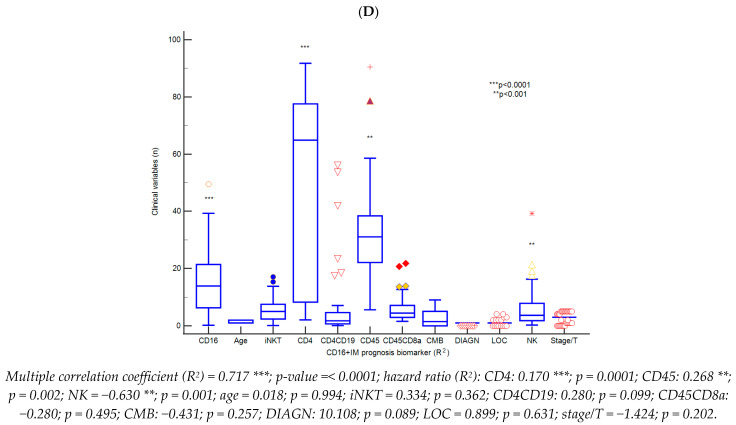
Receiver operating characteristic (ROC) analysis of CD-biomarkers (**A**,**B**) in sarcoidosis. (**C**,**D**) Prognostic biomarkers (CD56+CD16+NK; CD16+IM) in sarcoidosis. *** p < 0.0001, ** p < 0.001, and * p < 0.05 represent significant statistical differences between control and experimental samples by represented by Least squares multiple regression (R^2^) by MedCalc v20.111 Software Ltd., Ostend, Belgium. **Legend:** NKR—natural killer receptor; T—lymphocyte; AUC—area under the curve; PPV—positive value prediction; PNV—negative value prediction; CMB—comorbidities; DIAGN—diagnosis; LOC—localization; stage/T—associated treatment by sarcoidosis stage.

As a predictive model, the ROC curve for the CD56+CD16+ (NK) biomarker presents a discrimination threshold (AUC = 0.595 ± 0.095, *p* = 0.319) with modest accuracy, with high specificity for sarcoidosis diagnosis, represented by sensitivity of 31.03% and specificity of 100.00% (Figure 8A). The discrimination threshold (AUC = 0.543 ± 0.116, *p* = 0.709) for the CD16+CD56− (CD16IM) biomarker, with sensitivity of 96.55% and specificity of 25.00%, indicated modest accuracy and a non-specific biomarker in sarcoidosis diagnosis (Figure 8B).

CD56+CD16+ (NK) expression represents an independent predictive factor (R^2^ = 0.586; *p* = 0.018), positively correlated with iNKT (R^2^ = 0.723, *p* = 0.001) and negatively correlated with CD16 monocytes (R^2^ = −0.310, *p* = 0.003, Figure 8C). CD56+CD16+ represents a prognostic biomarker, being positively associated with disease progression and adverse outcomes (diagnosis: R^2^ = 7.474, *p* = 0.072) and negatively associated with the treatment response by sarcoidosis stage (R^2^ = −1.186, *p* = 0.128, Figure 8C).

As an independent predictive factor (R^2^ = 0.717; *p* < 0.0001) for sarcoidosis prognosis, CD16+CD56− intermediary monocytes (CD16+IM) were positively correlated with CD4+ T-helper lymphocytes (R^2^ = 0.170, *p* = 0.0001) and CD45+NKR (R^2^ = 0.268, *p* = 0.002) and negatively correlated with CD56+CD16+ NK cells (R^2^ = −0.630, *p* = 0.001, Figure 8D).

CD16+CD56− expression is a prognostic biomarker, being positively associated with disease progression (R^2^ = 10.108, p = 0.089) and negatively correlated with the treatment response by sarcoidosis stage (R^2^ = −1.424, p = 0.202, Figure 8D).

## 3. Discussion

CD biomarker analysis, yielding predictive models for diagnosis and prognosis, represents an important and innovative research tool for estimating disease progression and adverse outcomes in patients with different inflammatory responses across sarcoidosis stages.

Sarcoidosis granulomas are characterized by a macrophage syncytium surrounded by activated T cells. Sarcoidosis patients manifest various clinical symptoms. Incipient signs of acute sarcoidosis, such as cough, arthralgias, mediastinal and hilar lymphadenopathy, may disappear spontaneously. In resolved cases of sarcoidosis, granulomas involute and inflammation cease. In progressive sarcoidosis, granulomas persist and are maintained by chronic inflammation [26].

Macrophage plasticity is involved in the promotion and resolution of inflammation. In pulmonary sarcoidosis, alveolar macrophages produce IFN-γ, which stimulates granuloma formation. Classical activation of the M1 macrophage phenotype involves the release of pro-inflammatory cytokines such as IL-1β, IL-12, and TNF-α. Alternative activation of the M2 macrophage phenotype promotes tissue repair by releasing IL-4, IL-13, IL-10, and TGF-β. The role of M2 macrophages in fibrosis remains unclear, and corresponding studies are necessary to observe macrophage function in sarcoidosis and the transition from chronic granulomatous inflammation to fibrosis [9,27,28].

In our study, monocyte-derived macrophage activation (M1 phenotype), represented by CD8a+CD45+ (CD8aTCD45NKT) expression, showed higher values in granulomatous inflammation, in active and progressive sarcoidosis, being a predictive biomarker with very good accuracy and high specificity in establishing a diagnosis of sarcoidosis. As a prognostic biomarker, it is an independent predictor of disease progression, being negatively associated with applied treatments by sarcoidosis stage.

A biomarker used to establish a diagnosis of sarcoidosis in bronchoalveolar lavage (BAL) is a CD4/CD8 ratio > 3.5 accompanied by lymphocytosis > 15% [26]. Sarcoid granulomas are characterized by the accumulation of CD4+ and CD8+ T lymphocytes, B lymphocytes, monocytes, mast cells, fibroblasts, and mononuclear phagocytes [29,30,31]. Monocyte-derived macrophages are involved in tissue homeostasis and repair. Pathogen recognition receptors (PRRs) and Toll-like receptors (TLRs), with their phagocytic activity, have a surveillance function [32]. In sarcoidosis, a dysregulated Toll-like receptor response is involved in pathogenesis and clinical outcomes [33,34,35,36]. A classically activated M1 macrophage phenotype promotes granuloma formation, and the transition from an M1 to M2 macrophage phenotype during sarcoidosis progression leads to fibrosis [37]. An M2-like macrophage phenotype has been reported in sarcoid granulomas in the lungs, liver, heart, and lymph nodes [38,39]. CD14+ monocytes from circulation are differentiated into monocyte-derived DCs (moDCs) at inflammation sites. Monocyte-derived DCs regulate the responses of T cells by secreting pro-inflammatory chemokines at the inflammatory site [40,41].

Another biomarker that was analyzed was CD45+CD8a− (CD45NKR) expression, which showed moderate accuracy without specificity for sarcoidosis diagnosis. However, as a prognostic biomarker, it represents an independent predictive factor in sarcoidosis patients, being positively correlated with the applied treatment by sarcoidosis stage.

CD45NKR expression modulates dephosphorylation and segregation from the immune synapse. CD45NKR is involved in signaling responses, dependent on the target-cell engagement of the NK receptor ligand. The synergistic effect between CD45NKR and NK cells suggests a promising strategy to enhance their immunotherapeutic efficacy. Activated subpopulations of NK cells expressing the CD45NKR combination represent 80% of NK cells, whereas, separately, they constitute only 21–32% of NK cells [42,43,44,45].

Sarcoidosis is a systemic inflammatory disorder with a pathogenesis that remains incompletely understood. A sarcoidosis granuloma presents a central core with macrophage aggregates, epithelioid cells, and multinucleated giant cells, while T cells and dendritic cells (DCs) are present in the outer layer [45]. The granulomatous process is driven by an exaggerated immune response involving both the adaptive and innate immune systems. CD4+ T-helper lymphocytes are present in the outer layer of the granuloma. Circulating T lymphocytes, involved in active pulmonary sarcoidosis, express functional IL-2 receptors (IL-2R) [46,47,48]. Increased levels of IL-17A+ CD4+ T cells in blood samples indicate that Th17 lymphocytes are involved in granuloma formation [49].

In our study, CD19 monocyte-derived macrophages were characteristic of B cells, being involved in granuloma evolution as pro-inflammatory cells. CD4+ T-helper lymphocytes are responsible for the pro-inflammatory phenotype’s activation (M2 phenotype). The CD4+CD19+ biomarker exhibited a difference between granulomatous formation in active sarcoidosis in stage I–II and cases without granulomas in resolving sarcoidosis or non-active chronic sarcoidosis in stage I–II. CD4+CD19+ (CD4ThCD19M) expression as a biomarker had modest accuracy, without specificity for sarcoidosis diagnosis, being involved in the development and evolution of granulomas. As a prognostic biomarker, CD4+CD19+ expression is an independent predictive factor in sarcoidosis patients, being negatively correlated with the applied treatment by sarcoidosis stage.

Phenotypic changes in T cells in peripheral blood samples from patients with chronic sarcoidosis have led to the identification of new drug targets correlated with disease progression [14]. Increased levels of T-helper lymphocytes and greater CD4/CD8 lymphocyte ratios were reported in bronchoalveolar lavage (BAL) samples [12]. In sarcoidosis, CD4+ T-helper lymphocyte expression represents an adaptive immune response, because granuloma formation requires CD4+ cell activation [50]. In the lungs, CD4+ T-helper lymphocytes release cytokines such as IFN-γ, TNF-α, IL-12, and IL-18, which stimulate macrophages to form granulomas. The mediastinal lymph nodes are the initial sites for granuloma formation before the development of pulmonary granulomas [51]. Sarcoid granulomas present epithelioid and multinucleated giant cells, surrounded by CD4+ T-helper cells [52,53]. Granuloma initiation involves modifying the macrophage aggregate phenotype, which is necessary for transformation into epithelioid cells. CD4+ T-helper cells and inflammatory cytokines (IFN-γ, TNF-α, IL-12, IL-18) promote cell–cell interactions between macrophages and dendritic cells or monocytes, leading to the formation of multinucleated giant cells from the granuloma core, surrounded by T lymphocytes and B lymphocytes [54,55].

In sarcoid granulomas and lymph nodes, increased levels of Th1-polarized CD4+ cells and Th17 lymphocytes have been reported [56,57]. Th17 cells, a subgroup of CD4+ T lymphocytes, secrete IL-17A to induce IFN-γ and TNF-α production in macrophages, promoting giant-cell and granuloma formation [52,53]. Th17 cells exposed to TGF-β and the IL-6 signaling pathway determine the tyrosine phosphorylation of STAT3, inducing the activation of Th17-related genes such as IL-17A and IL-23R, as well as the transcription factor retinoic acid receptor-related orphan nuclear receptor γt (RORγt), a negative regulator of alternative lineage phenotypes [58,59].

Our study shows that the accumulation of activated CD4+ T-helper cells in sarcoid granulomas is responsible for the initiation of a pro-inflammatory phenotype. CD4+T-helper expression represents a biomarker with good accuracy and high specificity for sarcoidosis diagnosis. As a prognostic biomarker, in sarcoidosis patients, CD4+ T-helper expression is an independent predictive factor, being positively correlated with the applied treatment by sarcoidosis stage.

In sarcoidosis, T cells are specific biomarkers for cellular activation and are associated with a long-term prognosis. CD4+ stem cell-like memory (Tscm) expression was increased in patients with sarcoidosis, suggesting a favorable long-term prognosis, but an aberrant increase in activated Tscm indicates T-cell-mediated inflammation [60]. Aberrantly activated phenotypes of CD4+ T cells and macrophages are important in disease pathogenesis, suggesting their potential prognostic value in sarcoidosis [14,61]. Activated T cells, involved in neutrophil and monocyte recruitment, play an important role in their proliferation during granuloma formation. T and B lymphocytes are involved in the pathogenesis of sarcoidosis and granuloma formation. Increased B-cell expression was observed in granulomas and pulmonary lesions [62,63,64]. CD4+ T-helper (Th) cells can recognize and bind to MHC/oligopeptide complexes through T-cell receptors (TCRs) on their surfaces, forming an MHC/peptide/TCR complex, which plays a role in the activation signaling for antigen-specific T cells [65]. T-cell differentiation into effectors requires the release of IL-1β, IL-6, IL-12, IL-23, and transforming growth factor beta (TGF-β) from activated DCs and macrophages [13]. The immune response of activated T cells in granuloma formation involves the production of IFN-γ, IL-2, and IL-17A [66].

In sarcoidosis, increased expression of the chemokine receptor CXCR3 and the transcription factor T-bet represents a typical response of polarized Th1 cells [67]. Th17 cells contribute to sarcoid granuloma formation by expressing the Th17 chemokine receptor CCR6 in the mediastinal lymph nodes and by producing IFN-γ from CCR6- and CXCR3-co-expressing Th17.1 cells [13,57,68].

CD4+ T cells play an important role in the pathogenesis of sarcoidosis, as a polarized T-cell phenotype is observed in the lungs and lymph nodes of patients with sarcoidosis [69]. Naïve circulating T cells are differentiated into effector and memory T cells upon antigen recognition [70]. An increased level of activated naïve T cells in blood samples was correlated with a worse prognosis [13]. A highly activated CD4+ T-cell population was observed in patients with pulmonary sarcoidosis [22,56,71].

Our study showed that CD3+CD56+CD16+ (iNKT) expression has modest accuracy and high specificity for sarcoidosis diagnosis, but, as a prognostic biomarker, it is an independent predictor that is associated negatively with disease progression, being positively correlated with the treatment applied by sarcoidosis stage.

Various studies report that NKT cells are involved in the pathogenesis of sarcoidosis and granulomatous lesions [72,73,74]. In sarcoidosis, NKT expression was higher in stage I–II, indicating a negative correlation with T-lymphocyte expression [18]. In sarcoidosis, decreased iNKT-cell expression was observed in blood samples. In granulomatous lesions from patients with sarcoidosis, increased accumulation of iNKT cells [22,73] has been reported. Deregulated iNKT-cell function prolongs T-cell activity, a characteristic of the pro-inflammatory phenotype of sarcoidosis. In patients with sarcoidosis, a reduction in iNKT cells in peripheral blood and their absence from granulomas indicate the persistence of a T-cell immune response [75]. In sarcoidosis, reduced iNKT-cell counts in the blood lead to T-cell suppression by monocytes, resulting in an exaggerated immune response. Lower circulating iNKT-cell expression was associated with pulmonary fibrosis and disease severity [11,76].

In our study, the CD56+CD16+ (NK) biomarker presented modest accuracy, with high specificity for sarcoidosis diagnosis, but, as a prognostic biomarker, it is an independent predictor, being positively correlated with disease progression and negatively associated with treatment by sarcoidosis stage. Moreover, the CD16+CD56− (CD16IM) biomarker showed modest accuracy, without specificity for sarcoidosis diagnosis, being an independent predictive factor involved in sarcoidosis prognosis, positively correlated with disease progression and negatively associated with the applied treatment by sarcoidosis stage.

NK cells produce pro- and anti- inflammatory cytokines involved in the innate immune response [77,78,79]. In sarcoidosis, the presence of NK cell populations in bronchoalveolar lavage fluid (BALF) was associated with poorer outcomes and a higher likelihood of corticosteroid treatment. NK-cell counts in the peripheral blood (PB) were lower in sarcoidosis patients than in controls, with the lowest value observed in stage III [80]. Increased CD4+ T-helper and NK lymphocytes are present in the later stages of pulmonary sarcoidosis [81]. Bergantini et al. observed increased NK-cell expression in the peripheral blood of patients with sarcoidosis with subacute and chronic disease [20]. The observed negative correlation between NK cells and CD4+ T-helper lymphocytes indicated that sarcoidosis had progressed to an advanced pulmonary stage. Katchar et al. showed that NK cells, as indicated by increased CD56+ expression, were able to release cytokines [82]. NK cells accumulate in the lungs during advanced stages, contributing to the progression of sarcoidosis [81]. Natural killer (NK) cells are associated with poor outcomes. Activated NK cells stimulate TNF-α secretion from alveolar macrophages, leading to progressive, corticosteroid-resistant sarcoidosis. Deregulated pulmonary function is associated with the increased accumulation of NK cells in the lungs [80]. NK cells (CD16+CD56+) play an important role in immune defense and are involved in the pathogenesis of inflammatory pulmonary diseases [20]. NK cells can release various cytokines, including TNF-α, IL-4, IL-10, IL-13, IL-17, and IL-21 [23].

Because monocytes are precursors of macrophages, they play an important role in sarcoidosis. Intermediate monocytes (CD14+CD16+) exhibit pro-inflammatory phenotypes in sarcoidosis [83]. Prednisone and infliximab treatments used in sarcoidosis downregulate intermediate monocyte activity, suggesting a prognostic biomarker correlated with treatment response [84,85]. Various circulating monocyte populations are of interest as prognostic biomarkers [86]. In active sarcoidosis, activated monocytes in blood samples exhibited increased CD16 expression [85].

Natural killer (NK) cells secrete pro- and anti-inflammatory cytokines, indicating different phenotypes. In patients with sarcoidosis, the accumulation of CD56+CD16+ (NK cells) was reported in BALF and blood samples [82]. An increased NK cell count in BALF samples from patients with sarcoidosis was associated with poorer outcomes and a higher likelihood of corticosteroid treatment [80]. Natural killer cells and T cells play a role in protecting against cancer [87,88]. In sarcoidosis, CD56+CD16- expression produced elevated TNF-α and IFN-γ levels in BALF samples compared to healthy controls and was involved in an aberrant immune response and T-cell activation via the Janus kinase (JAK) signaling pathway [89]. Understanding the T-cell phenotypes involved in progressive sarcoidosis may lead to the discovery of new therapies to target it. Increased CD14+CD16+ intermediate monocytes have been associated with the development of chronic sarcoidosis [90].

The limitations of this study are represented by the specificity of the biomarkers, because sarcoidosis is a heterogeneous disorder and not a typical form of inflammation, as well as the low number of patients with sarcoidosis. Biomarkers with optimal accuracy and specificity in sarcoidosis are limited, but the possibility to study specific biomarkers involved in different stages of sarcoidosis could lead to future work highlighting the immune mechanisms underlying sarcoidosis phenotypes and the development of promising tools to advance sarcoidosis research.

## 4. Materials and Methods

### 4.1. Case Selection

Blood samples (*n* = 64) were obtained from patients (who signed informed consent forms, agreeing to participate in the study) who were being treated in the Clinical Department of Pneumology within the Pneumophthisiology Hospital of Constanta, Romania. Patients with sarcoidosis were treated in various centers associated with this hospital. Only diagnosed patients with sarcoidosis in the hospital were used for the identification of new and alternative CD biomarkers from blood samples, using rapid and standard laboratory techniques such as flow cytometry, at the Cell Biology Department of the Centre for Research and Development of Morphological and Genetic Studies of Malignant Pathology, Ovidius University of Constanta, Romania (CEDMOG).

Patients were divided into four groups: (A) active sarcoidosis, granulomatous inflammation, stage I–II (*n* = 26); (B) resolving sarcoidosis or non-active chronic sarcoidosis, without granulomatous inflammation, stage I–II (*n* = 18); (C) progressive sarcoidosis, active chronic sarcoidosis, granulomatous inflammation, stage II–III (*n* = 12); (D) healthy patients (*n* = 8).

### 4.2. Clinical Aspects

The detection of pulmonary sarcoidosis in the Pneumology Department within the Pneumophthisiology Hospital of Constanta, Romania involves a detailed clinical evaluation, a physical examination, and various imaging and functional tests. Chest radiography and computed tomography are used to visualize the lungs and identify granulomas. Pulmonary function tests measure lung capacity and function, and biopsy may be necessary to confirm the presence of granulomas. The diagnosis of pulmonary sarcoidosis is based on a compatible clinical and imaging presentation, the absence of granulomas on histological examination, and the exclusion of other causes of inflammation.

The classification of sarcoidosis patients by localization was as follows: (1) active sarcoidosis, mediastinal–pulmonary (*n* = 24) and pulmonary and lymph node involvement (*n* = 2), stage I–II; (2) resolving sarcoidosis or non-active chronic sarcoidosis, mediastinal–pulmonary (*n* = 14); mediastinal, pulmonary, hepatic, and splenic (multi-system, *n* = 2); and mediastinal, cutaneous, and renal (multi-system, *n* = 2), stage I–II; (3) progressive sarcoidosis, active chronic sarcoidosis, mediastinal–pulmonary (*n* = 12), stage II–III.

### 4.3. Reagents and Equipment

Our study used a flow cytometer (Attune, Acoustic Focusing Cytometer, Applied Biosystems, part of Life Technologies, Waltham, MA, USA). The flow cytometer was set using fluorescent beads (Attune performance tracking beads, labeling, and detection, Life Technologies, Europe BV, Bleiswijk, The Netherlands) with a standard size (bead populations with four intensity levels). The quantity was established by enumerating cells below 1 µm, with 10,000 cells per sample for each analysis, gated by forward scatter (FSC) and side scatter (SSC) [91]. The density plot was split into four quadrants to determine the single-positive population for each biomarker and both double-negative and double-positive populations. Our flow cytometry graphs display two measurement parameters—one on the x-axis and one on the y-axis—with the events shown as density plots (two-parameter density plot method). Quadrants are placed at different points because cell populations are interpreted as a function of the negative control histogram (mouse IgG1 kappa isotype PE) overlaid onto the stained positive dataset, allowing positive cells to be accurately identified.

The second method of gathering flow cytometry data was represented by graphs displaying two measurement parameters—one on the x-axis and one on the y-axis—and the events from the dot plot are quantified by placing gates around distinct populations. For this method, three references were added: a mouse IgG1 kappa isotype PE (IgG1) as a negative control and C2 and C27 control samples recovered from healthy patients to increase the accuracy of the method. Cell populations were measured and interpreted using the Attune Cytometric Software v.1.2.5, 2010, Applied Biosystems, Waltham, MA, USA.

Anti-Hu CD45- FITC (Clone HI30, eBioscience, Invitrogen, Life Technologies Corp., Carlsbad, CA, USA), anti-Hu CD8a− PE (Clone RPA-T8, eBioscience, Invitrogen, Life Technologies Corp., Carlsbad, CA, USA), anti-Hu CD4- Alexa Fluor 488 (Clone OKT4, eBioscience, Invitrogen, Life Technologies Corp., Carlsbad, CA, USA), and anti-Hu CD19− PE (Clone H1B19, eBioscience, Invitrogen, Life Technologies Corp., Carlsbad, CA, USA) were used to assess T-cell activation and the inflammation status, represented by T-helper lymphocyte and B-cell expression. Anti-Hu CD3- Alexa Fluor 488 (Clone OKT3, eBioscience, Invitrogen, Life Technologies Corp., Carlsbad, CA, USA), anti-Hu CD16 PE-Cyanine7 (Clone eBioCB16, eBioscience, Invitrogen, Life Technologies Corp., Carlsbad, CA, USA), and anti-Hu CD56− PE (Clone TULY56, eBioscience, Invitrogen, Life Technologies Corp., Carlsbad, CA, USA) were used to show NK-cell, monocyte, and T-cell expression.

### 4.4. CD 45-FITC/CD8a−PE, CD 4- Alexa Fluor 488/CD19− PE, and CD3- Alexa Fluor 488/CD16 PE-Cyanine7/CD56− PE Flow Cytometry Analysis

Blood samples were collected into tubes (4 mL) with anticoagulant EDTA-K2. Flow cytometry tubes with blood samples were analyzed as follows: (1) CD 45-FITC and CD8a−PE; (2) CD 4- Alexa Fluor 488 and CD19− PE; (3) CD3- Alexa Fluor 488, CD16 PE-Cyanine7, and CD56− PE; (4) negative control—mouse IgG1. To observe the differences between experimental and control samples, an antigen–antibody reaction was used. In flow cytometry tubes, 100 µL of the blood sample was mixed with 5 µL of a different CD biomarker; the mixture was then vortexed and incubated in the dark for 25 min at 37 °C. Then, 1 mL of flow cytometry staining buffer (FSB) was added to each tube, which was then vortexed for 1 min before analysis. NK cells, monocytes, and T cells were identified by flow cytometry analysis based on size and CD staining.

### 4.5. Statistical Analysis

CD8a+CD45+ (%; CD8aTCD45NKR), CD45+CD8a− (%; CD45NKR), CD4+CD19+ (%; CD4ThCD19M), CD4+CD19− (%; CD4Thelper), CD3+CD56+CD16+ natural killer T cell (%; iNKT), CD56+CD16+ natural killer cell (%; NK), and CD16+CD56− intermediary monocyte (%; CD16IM) expression was reported as the mean ± standard deviation (SD). The Mann–Whitney test via the MedCalc v20.111 Software Ltd., 2010 (Ostend, Belgium) was used to compare the means between the three experimental groups and controls, assuming normal distributions. Differences between control and experimental samples with *p* < 0.05 were considered statistically significant.

Receiver operating characteristic (ROC) analysis using the MedCalc v20.111 Software Ltd., 2010, Ostend, Belgium was used to assess the accuracy of CD biomarkers for sarcoidosis diagnosis. The sensitivity and specificity of the CD biomarkers were calculated using the Youden index (optimal cut-off point), which corresponds to the maximum area under the curve (AUC). Accuracies in terms of optimal positive value prediction (PPV) and negative value prediction (PNV) were calculated in relation to the true positive rate (TPR = sensitivity) and false positive rate (FPR = 100 − specificity) via the ROC curves.

Prognostic CD biomarkers in sarcoidosis were represented by least-squares multiple regression (R^2^) using the MedCalc v20.111 Software Ltd., 2010, Ostend, Belgium. Differences between variables with *p* < 0.05 were considered statistically significant. Figure 1, Figure 3, Figure 5 and Figure 7 were generated using the Attune Cytometric Software v.1.2.5, Applied Biosystems, 2010 (Bedford, MA, USA). Figure 2, Figure 4, Figure 6 and Figure 8 were generated using the MedCalc v20.111 Software Ltd., 2010 (Ostend, Belgium).

## 5. Conclusions

Given the heterogeneous nature of sarcoidosis, our study highlights biomarker discovery for diagnosis and prognosis. Monocyte-derived macrophage activation (M1 phenotype), represented by CD8a+CD45+ (CD8aTCD45NKR) and CD4+CD19− (CD4 T helper lymphocytes, M2 phenotype), is a specific biomarker involved in the establishment of a sarcoidosis diagnosis.

The levels of CD8a+CD45+, CD3+CD56+CD16+ (iNKT), CD56+CD16+ (NK), and CD16+CD56− (CD16IM), as prognostic biomarkers, represent independent predictors of disease progression in relation to the applied personalized treatment.

## Figures and Tables

**Table 1:** Summary of clinical and demographic data of patients collectively and divided into three study groups.

No.	Group/ Parameter	(A) Active Sarcoidosis, Granulomatous Inflammation, Stage I–II (SI_IIG; *n* = 26)	(B) Resolving Sarcoidosis or Non-Active Chronic Sarcoidosis, Without Granulomatous Inflammation, Stage I–II (SI-II Cr; *n* = 18)	(C) Progressive Sarcoidosis, Active Chronic Sarcoidosis, Granulomatous Inflammation, Stage II–III (SII_SIIIG; *n* = 12)	(D) Healthy Patients (C; *n* = 8)
1.	Age	50.12 ± 12.71	57.50 ± 11.49	58.20 ± 16.48	47.50 ± 4.03
2.	Localization	Mediastinal–pulmonary (*n* = 24); pulmonary and lymph node involvement (*n* = 2).	Mediastinal–pulmonary (*n* = 14); mediastinal, pulmonary, hepatic, and splenic (multi-system, *n* = 2); Mediastinal, cutaneous, and renal (multi-system, *n* = 2).	Mediastinal pulmonary (*n* = 12).	
3.	Treatment	Corticosteroid (*n* = 4); without treatment (*n* = 22).	Corticosteroid (*n* = 2); without treatment (*n* = 16).	Without treatment (*n* = 12).	
4.	Comorbidities	Bronchial asthma, stationary adenopathy, bronchiectasis, exertional angina pectoris (*n* = 4); chronic respiratory failure (*n* = 2); obesity grade II (*n* = 4); HTAE (*n* = 8); acute lingering bronchitis (*n* = 2).	Hypothyroidism (*n* = 2); type II diabetes mellitus (*n* = 2); HTAE (*n* = 4); latent chronic respiratory failure (*n* = 2); chronic peripheral venous insufficiency (*n* = 2).	Mild mitral insufficiency, grade II aortic insufficiency (*n* = 2); HTAE (*n* = 2); interstitial pulmonary fibrosis (*n* = 2); HTAE, obesity grade III (*n* = 6).	
5.	CD8aTCD45NKR (CD45+CD8a+)	6.09 ± 3.21 **	4.08 ± 2.19	6.29 ± 3.00 *	2.75 ± 0.58
6.	CD45NKR (CD45+CD8a−)	32.54 ± 11.71	32.17 ± 13.42	24.00 ± 11.37	42.79 ± 26.39
7.	CD4ThCD19M (CD4+CD19+)	2.86 ± 2.19	0.91 ± 1.00	7.88 ± 11.55	6.26 ± 9.18
8.	CD4Thelper (CD4+CD19−)	74.05 ± 12.61 **	7.54 ± 3.30 **	56.83 ± 34.60 **	45.86 ± 28.24
9.	iNKT (CD3+CD56+CD16+)	5.07 ± 3.47	3.33 ± 2.80	6.10 ± 3.16	6.71 ± 5.26
10.	NK (CD56+CD16+)	5.29 ± 4.97	7.26 ± 9.55	4.26 ± 3.71	6.44 ± 5.81
11.	CD16IM (CD16+CD56−)	20.74 ± 11.84 *	8.19 ± 8.28	11.88 ± 6.84	12.82 ± 9.32

**Note:** ** *p* < 0.01 and * *p* < 0.05 indicate statistically significant differences between control and experimental samples via Mann–Whitney test using the MedCalc software, Ostend, Belgium. **Legend:** NKR—natural killer receptor; T—lymphocyte; Th—T-helper lymphocyte; M—monocyte; iNKT—natural killer T cell; NK—natural killer; IM—intermediary monocyte; HTAE—hypertension.

## Data Availability

The original contributions presented in this study are included in the article. Further inquiries can be directed to the corresponding author.

## Abbreviations

The following abbreviations are used in this manuscript: CD45NKRCD8aT = CD45+CD8a+; CD45NKR = CD45+CD8a−; CD4ThCD19M = CD4+CD19+; CD4Thelper = CD4+CD19−; iNKT = CD3+CD56+CD16+; NK = CD56+CD16+; CD16IM = CD16+CD56−; Th1—T-helper 1; DCs—dendritic cells; NK—natural killer cells; NKT—natural killer T cells; NKR—natural killer receptor; T—lymphocyte; IM—intermediary monocytes; AUC—area under the curve; PPV—positive value prediction; PNV—negative value prediction; stage/T—associated treatment by sarcoidosis stage; C—control samples recovered from healthy patients; SI_SIIG—active sarcoidosis, granulomatous inflammation, stage I–II; SI_SIICr—resolving sarcoidosis or non-active chronic sarcoidosis, without granulomatous inflammation, stage I–II; SII_SIIIG—progressive sarcoidosis, active chronic sarcoidosis, granulomatous inflammation, stage II–III; BAL—bronchoalveolar lavage; RORγt—transcription factor retinoic acid receptor-related orphan nuclear receptor γt; Tscm—CD4+ stem cell-like memory; TGF-β—transforming growth factor beta; TCR—T-cell receptor; PB—peripheral blood; JAK—Janus kinase signaling pathway.
